# Antibiotic Resistance in Vibrio Bacteria Associated with Red Spotting Disease in Sea Urchin *Tripneustes gratilla* (Echinodermata)

**DOI:** 10.3390/microorganisms12122460

**Published:** 2024-11-29

**Authors:** Mayan Ben Natan, Matan Masasa, Nadav Shashar, Lior Guttman

**Affiliations:** 1 Marine Biology and Biotechnology Program, Department of Life Sciences, Ben-Gurion University of the Negev, Eilat Campus, Eilat 8855630, Israel; mayanze5@gmail.com (M.B.N.); nadavsh@bgu.ac.il (N.S.); 2 Israel Oceanographic and Limnological Research, The National Center for Mariculture, P.O. Box 1212, Eilat 8811201, Israel; masasam@post.bgu.ac.il; 3 Department of Life Sciences, Ben-Gurion University of the Negev, Be’er Sheva 8410501, Israel

**Keywords:** antibiotic resistance genes, aquaculture, multi-drug tolerance, red spotting disease, sea urchins, Vibrio

## Abstract

The red spotting disease harms sea urchins to the extent of mass mortality in the ocean and echinocultures, accompanied by environmental damage and economic losses. The current study emphasizes the antimicrobial resistance of three isolated bacteria, closely related to *Vibrio harveyi*, *Vibrio owensii*, and *Vibrio fortis*, associated with red spotting in the cultured sea urchin *Tripneustes gratilla*. In vitro trials examined the susceptibility of these bacterial isolates to various antibiotics. In addition, using an in silico examination, we revealed the arsenal of antimicrobial resistance genes in available genomes of various pathogenic *Vibrio* associated with diseases in sea urchins, fish, shellfish, and corals. These two approaches enabled the discussion of the similarities and differences between aquatic pathogenic *Vibrio* and their antibiotic resistance. Among them, we revealed a core resistance to tetracyclines and penams by the in vitro examined strains. At the same time, the in silico study also supported this core resistance by the presence of the *adeF* and *CRP* genes in the bacterial genomes. Nevertheless, variability and specific resistance were evident at the species and strain levels in the *Vibrio* bacteria and genomes. The in vitro trials highlighted the diverse resistance of the *Vibrio harveyi*-like isolate to all examined antibiotics, while the other two isolates were found susceptible to nitrofurantoin and sulfamethoxazole. The resistance of the *Vibrio harveyi*-like isolate could not have been obtained in the genome of the proposed relative of *Vibrio harveyi* VHJR7 that lacks the *oqxA* and *oqxB* genes, which enables such a resistance. A unique sensitivity of the *Vibrio fortis*-like isolate to erythromycin is proposed when compared to other isolated *Vibrio* and *Vibrio* genomes that seem capable of resisting this drug. According to the results, we propose nitrofurantoin or sulfamethoxazole for treating two of the red-spotting-associated isolates (*Vibrio fortis* and *Vibrio owensii*-like), but not *Vibrio harveyi*-like. We assume that a shared resistance to some antibiotics by Vibrios is gained by a horizontal gene transfer while previous exposures of a bacterial strain to a specific drug may induce the development of a unique resistance. Finally, we discuss the novel knowledge on antibiotic resistance in *Vibrio* from the current research in light of the potential risks when using drugs for disease control in aquaculture.

## 1. Introduction

**Figure 1:**
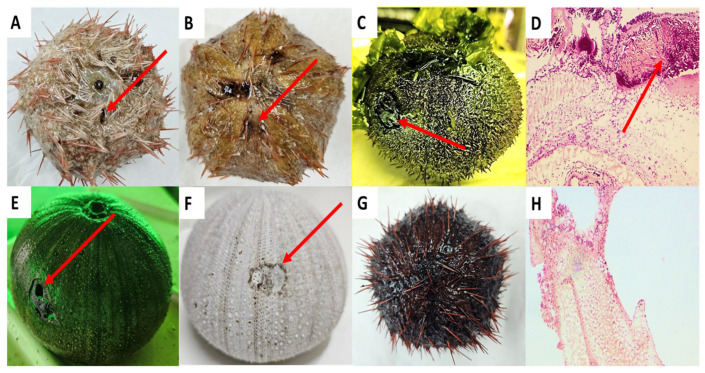
Clinical symptoms of the red spotting in *T. gratilla* at NCM, demonstrating disease development in infected sea urchins, as compared to healthy individuals. (**A**) Loss of spines and occurrence of small red dots; (**B**) red dots expand to patches as spines continue to fall; (**C**) local lesion damaging the skeleton; (**D**) histological section of the damaged skeleton; (**E**) lesion creates hole in the skeleton in dying individual; (**F**) dead individual with the harmed skeleton; (**G**) healthy *T. gratilla*; and (**H**) healthy tissue of a healthy sea urchin. Red arrows indicate red dots and the infected tissue.

Red spotting is a common disease that causes the mass mortality of sea urchin populations in their natural habitats or echinocultures worldwide [1,2]. The disease is characterized by the loss of spines followed by the occurrence of red–purple dots on the outer skeleton where spines have been detached. The spine loss continues, accompanied by the further development of the red–purple dots into larger patches and plaques. As the disease further develops, these patches and lesions are further spread to other parts on the outer skeleton and cause similar damages while leading to a severe loss of the outer organs, holes in the skeleton, and finally to death [1,2,3] (Figure 1). Such events involve heavy environmental damages and economic losses, while some of the damages are considered irreversible. Until now, studies on red spotting are relatively negligible and seem to focus on the economically valuable purple sea urchin *Strongylocentrotus intermedius*, mainly from farms in China [1,3]. The collector sea urchin, *Tripneustes gratilla*, is an important player in the marine environment, while it also has a high economic value, which encourages its cultivation worldwide. Nevertheless, a knowledge gap exists concerning pathogens that infect this sea urchin with red spotting and the potential efficacy of antibiotics in mitigating such pathogens in echinocultures.

Among aquatic pathogens, the bacterial genus *Vibrio* is infamous since many members of the taxon present a broad virulence and are responsible for various diseases of sea urchins, fish, shellfish, and corals [4,5]. Diseases include vibriosis, lesions, spotting, gastro-enteritis, muscle necrosis, tail rot, white tails, and a black shell, and have been documented in various aquaculture setups and conditions. This makes the mitigation of pathogenic *Vibrio* from the culture challenging while using antimicrobials as the primary strategy toward this goal. Such a strategy comes with several costs that are associated with the development of antimicrobial-resistant bacteria (ARBs), which are a major concern in public health on a global scale. Antimicrobial resistance (AMR) is often carried by a virulent bacterium, but can be spread forward to other bacteria through water, air, soil, or the host animal or plant. The mechanisms by which AMR is distributed include cell conjugation, transduction, or the horizontal gene transfer (HGT) of mobile genetic elements (MGEs) in the form of transposons or plasmids [6,7]. The aquatic environment presents an extensive reservoir of antimicrobial-resistant bacteria (ARBs) that carry antimicrobial-resistant genes (ARGs), which can be transferred through the food chain to humans [8]. Over recent decades, aquaculture has intensified significantly and is currently the primary supplier of animal protein for human consumption. The rapid growth is accompanied by an expanded use of antibiotics to control disease outbreaks [9,10]. The standard antibiotic treatments involve the therapeutic administration of a specific compound following the identification of the disease-causing agent or prophylactic administration of the compound at a sub-therapeutic level to prevent the succession of such an agent. However, the inappropriate use of antibiotics may involve administering several antibiotics simultaneously to reduce the risk of pathogen outbreaks or using unsuitable doses [11]. Moreover, the permanent presence of antibiotic residues is familiar in routine use [12]. According to a recent study, much of the administrated dosage of antibiotics, between 70 and 80%, is unretained by cultured animals and released into the water [10]. It is why prophylactic treatment with antibiotics has come under criticism, as it may reduce the probability of disease outbreaks, but it increases the probability of ARB development and human exposure to the antibiotic residues in the edible seafood produced [13,14]. Moreover, the permanent background levels of the unretained antibiotics increase the probability for ARB development in the culture water and cultivated organism and, with that, a greater chance for the occurrence of new virulent ARBs and their further spread [15,16].

In *Vibrio*, AMR is broad and ranges from bacteria with resistance to a sole drug to such with multiple-drug resistance (MDR). Recently, the reviewed data on *Vibrio* bacteria from the fish and shellfish mariculture revealed that a general susceptibility is uncommon, meaning that many of the isolated bacteria are resistant to either a single or several bioactive compounds [17,18,19,20]. Among the tested antibiotics in the different studies, resistance to ampicillin and streptomycin is common in *Vibrio* isolates from seabass and turbot cultures [17,21], while resistance to tetracycline, quinolones, and streptomycin is frequent in *Vibrio* from salmon cultures [22,23]. In shrimp cultures, *Vibrio* resistance to ampicillin, cefoxitin, chloramphenicol, erythromycin, oxacillin, streptomycin, sulphonamide, and tetracycline is common [18,20,24,25]. Various strains of *V. harveyi* have shown MDR, but an interspecific dissimilarity was evident when utilizing the type of compounds to which different strains are resistant [19]. A survey of different strains of *V. harveyi* from disease outbreaks in marine fish cultures proposed a shared MDR against ampicillin, amoxicillin, and cefoxitin. At the same time, such shared immunity was also evident in different strains of *Vibrio parahaemolyticus* and *Vibrio vulnificus* [26]. *Vibrio splendidus* and *Vibrio tasmaniensis* revealed MDR to chloramphenicol, oxytetracycline, and ampicillin [27], while many of the strains in each of the bacterial taxa of *Vibrio alginolyticus*, *V. harveyi*, *V. vulnificus*, or *V. parahaemolyticus* were resistant to penicillin, carbenicillin, ampicillin, cefalotin, and kanamycin [28,29]. Other strains of *V. alginolyticus* and *V. harveyi* were resistant to sulfamethoxazole and cefalotin [28,29].

Concerning the above, much of the existing data on antibiotic resistance in mariculture-associated *Vibrio* have been generated from studies of diseases of valuable fishes like salmon, rainbow trout, turbot, and yellowtail, and valuable shrimps like *Penaeus monodon*, *Penaeus japonicus*, and *Sicyonia ingentis* [19,20]. Data concerning the phenomenon in bacterial pathogens of other cultured invertebrates are relatively negligible. They mainly arrive from surveys on the infected organisms after arriving at the seafood market rather than in the culture environment [30,31,32]. Although *Vibrio* bacteria have been associated extensively with sea urchin's diseases (Table 1), knowledge gap concerning Vibrio pathogens and their antibiotic resistance has driven the current research. That said, the current research aimed at identifying bacterial pathogens associated with red spotting disease in *T. gratilla* and characterizing their resistance to various drugs that are commonly used in aquaculture. In addition, we aimed to compare results on antibiotic resistance between the red-spotting-associated pathogens and other pathogenic Vibrio that harm aquatic organisms. The hypothesis of the current research was that pathogens associated with red spotting disease in an echinoculture can resist diverse drugs.

## 2. Materials and Methods

### 2.1. Identification of Red-Spotting-Infected Sea Urchins

Red spotting disease in the echinoculture facility at the National Center for Mariculture in the Gulf of Aqaba, Eilat, Israel (NCM), was first observed in August 2021. The echinoculture facility is located in a greenhouse and consists of ~600 individuals of *Tripneustes gratilla elatensis* spread over 6 U-shaped polyvinyl chloride tanks (5.3 × 0.60 × 0.3 m) [42]. The echinoculture facility at the NCM is part of an integrated multi-trophic aquaculture system (IMTA) consisting of sea urchin tanks, three fishponds for the culture of sea bream (*Sparus aurata*), and one culture pond for seaweed *Ulva fasciata* that also functions as a biofilter for the discharged effluent, as described elsewhere [42]. The echinoculture receives fresh seawater from the Gulf of Aqaba, pumped from about 300 m off-shore at a depth of 13 m (320°29′ N and 580°34′ E), which is collected into a 10 m^3^ header tank and then routed to the urchin culture tanks. The seawater flow rate to the sea urchin culture tanks is set at 5 m^3^ h^−1^, recommended for maintaining high-quality water [43]. Red spotting was diagnosed independently by several experts. The first was the expert aquatic pathobiologist at the NCM (Dr. Galit Sharon, DVM), the second was the pathobiology research team at the NCM (led by Dr. Rosa Strem), and the third was the local research assistant at the NCM, Mr. David Ben-Ezra, all of whom havin over 30 years of experience in sea urchins culture and research. In addition, at the same time of appearance in the echinoculture, the occurrence of red spotting was identified also in the wild population of *T. gratilla* in the coastal area of the Gulf of Eilat by staff members of the National Monitoring Program of The Gulf of Eilat and the Israeli National Parks Authority. The clinical symptoms of red spotting are unique compared to other diseases. Foremost, the appearance of red–purple spots on the skeleton, which is identified right after the detachment of spines from the infected area. The red–purple dots develop into red–purple patches and plaques which are further spread to new parts on the skeleton until the tissue is totally damaged and a hole appears in the skeleton, leading to the animal’s death (Figure 1). Since the discovery of red spotting in the echinoculture, the rapid monitoring of the animals and their culture environment has taken place, accompanied by the sampling and examining of the obtained infected animals and their tissues, as compared to healthy individuals and tissues.

### 2.2. Bacterial Isolation and Identification

Following the diagnosis of red-spotting-infected sea urchins, infected and healthy tissues were sampled from infected and healthy individuals. Infected tissues were sampled from 10 infected individuals while for each individual, an additional tissue sample was taken from a distant, non-infected area on the skeleton. In addition, 6 healthy sea urchins were randomly sampled for healthy tissues. While aiming to identify the primary pathogen associated with red spotting, infected samples were such that exhibited the early signs of the disease, i.e., a small red–purple spot. All tissue samples were collected by a gentle scrubbing of the sampled area using sterile swab sticks. The sampled tissues were sown immediately on agar plates with a medium of thiosulfate citrate bile salts (TCBS). All developed colonies were transferred to new, similar agar plates until an isolated colony was observed. Following this procedure, isolated colonies were sampled for DNA extraction using the PureLinkTM Microbiome DNA Purification Kit (Thermo Fisher Scientific, Waltham, MA, USA) according to the manufacturer’s protocol. The DNA extracts were further transferred to amplify a targeted region in the 16SrRNA gene using a polymerase chain reaction (PCR) and the recommended primers set of 515F (GTGYCAGCMGCCGCGGTAA) and 926R (CCGYCAATTYMTTTRAGTTT). The amplification process included denaturation at 95 °C for 5 min, 28 cycles at 94 °C for 45 s, 50 °C for 60 s, 72 °C for 90 s, and a final elongation at 72 °C for 10 min. PCR products were run on an agarose gel to verify the length of the amplified sequence and sent for Sanger sequencing (sequencing with reverse and forward primers) at a certified sequencing lab (Hy-Labs Ltd., Rehovot, Israel). The sequencing results of both the reverse and forward primers were analyzed using BLAST software (https://blast.ncbi.nlm.nih.gov/Blast.cgi, accessed on 28 November 2024). Forward and reverse sequences were aligned while confirming their matching and further analyzed to identify their closest relatives. A similar bacteria isolation and identification procedure was performed using tissue scrubs from non-infected regions on the skeleton. Bacterial isolates that were found in both the infected and healthy tissues were sorted out to continue the research on the bacterial isolates that were present only in the infected tissue while not present in any of the samples of the healthy tissue. The 16S rRNA gene sequences of three bacterial isolates that were assumed to be pathogens in red spotting disease were deposited in the GenBank database under the respective accession numbers HQ449976.1, MT510177.1, and MT510178.1.

### 2.3. In Vitro Antibiotics Susceptibility Assay

The susceptibility of the isolated *Vibrio* strains to antibiotics was measured in vitro using the Kirby–Bauer disc diffusion method [44]. The following are the examined antibiotics and their doses (amount is per disc): amoxicillin (30 µg), ampicillin (10 µg), tetracycline (30 µg), erythromycin (15 µg), nitrofurantoin (300 µg), and sulfamethoxazole (25 µg). These antibiotics were selected because aquaculturists commonly use them for disease control and prevention [45]. Moreover, their target sites and activity mechanisms are also different [46]. The assay allows us to assess the extent to which a given bacterium resists antibiotic treatment and each compound’s feasibility and relative efficacy for controlling different *Vibrio* strains. The assay was performed separately for each bacterium and drug in triplicates. This means that in each trial, one of the bacteria was examined in three different agar plates (as triplicates) that consisted of a specific drug. In each plate, a bacterial colony was sown uniformly on the TSA agar surface and exposed to a Kirby–Bauer disc that was submerged in advance in a ready solution with the tested antibiotic compound. This allowed the diffusion of the harmful compound into the agar surface where bacteria grow. Following this procedure, the plate was transferred to incubation at 24 °C for 24–48 h. As a control, the same procedure was conducted using a similar set of plates (in triplicates) that consisted of the examined bacterium, but with an antibiotic-free disc. Following incubation, the diameter of the zone of inhibition was measured to evaluate the efficacy of each antibiotic in inhibiting bacterial growth and the sensitivity of the isolate to the antibiotic. The sensitivity rate of the bacterium to the antibiotic was classified according to the growth inhibition zone and rated as either sensitive (S), intermediate (I), or resistant (R). The intermediate susceptibility values in the diameter of the inhibition zone for the antibiotics ampicillin, tetracycline, erythromycin, nitrofurantoin, and sulfamethoxazole were defined both under the interpretive criteria standards by the Clinical and Laboratory Standards Institute (CLSI, 2012, [47]) and following the recommended values [48] for the antibiotic amoxicillin as follows: Amoxicillin: 19–20 mm; Ampicillin: 16–17 mm; Tetracycline: 15–18 mm; Erythromycin: 14–22 mm; Nitrofurantoin: 15–16 mm; and Sulfamethoxazole: 11–15mm. A bacterium was defined as either sensitive or resistant to the examined antibiotic if the measured diameter of the inhibition zone was extended or shortened, respectively, compared to the defined value for intermediate susceptibility.

### 2.4. In Silico Survey of Antibiotics Resistance in Vibrio spp.

A genomic survey of antibiotic resistance genes was performed on 14 *Vibrio* bacteria of different species that have been identified as aquatic pathogens and have had their genome deposited in the Genbank database, as detailed in Table 2. Among the analyzed genomes, five are pathogenic *Vibrio* spp. that harm sea urchins, while the other nine are pathogenic *Vibrio* spp. that harm fish, crustaceans, or corals (Table 2). Concerning the here-isolated *Vibrio* bacteria, a genomic analysis was performed using genomes of pathogenic *Vibrio* with an identical 16S rRNA gene sequence, as confirmed by Basic Local Alignment Search Tool analysis (BLAST). All genomes were analyzed for their content of antibiotic resistance genes using the web platform of the comprehensive antibiotics resistance database (CARD) and the resistance genes identifier (RGI) tool kit.

## 3. Results

### 3.1. Potential Vibrio Pathogens Associated with Red Spotting Disease in T. gratilla

**Figure 2:**
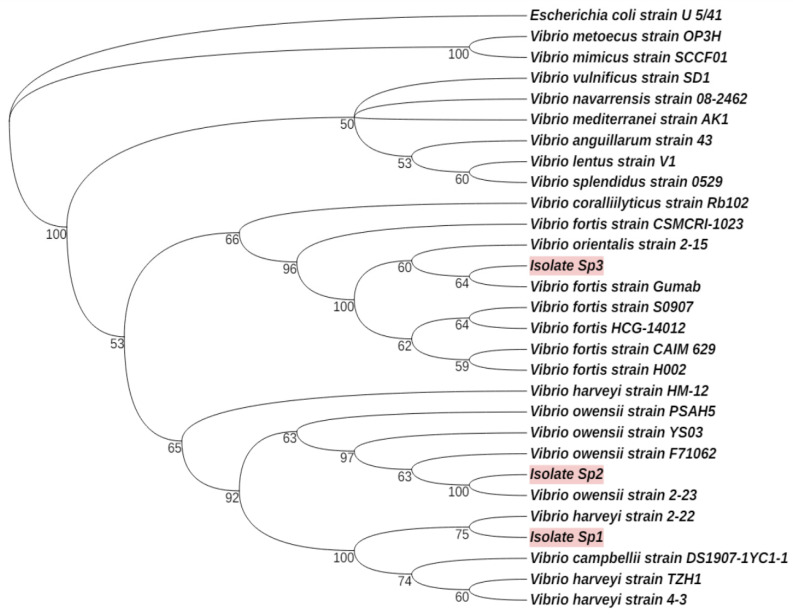
A phylogenetic tree of the relative closeness of the potential *Vibrio* pathogens that were isolated from infected sea urchins (marked in red) to other members of *Vibrio*, based on results from the 16SrRNA gene sequencing. The phylogenetic tree was generated using the online MEGA11 and iTOL tools. Relative closeness was determined following UPGMA and bootstrap analyses over 1000 trials. *E. coli* U 5/41 was set as an outer group. The value on each branch represents the percentage of correspondence to the phylogenetic branch kinship.

Bacterial enrichment resulted in the isolation of three potential pathogens in red spotting. The isolated bacteria were considered as such because they were present only in the infected tissues and not in healthy tissue samples. The BLAST analysis of the sequenced region in the 16S rRNA gene of these bacteria revealed the first isolate (here referred to as *Vibrio* sp. 1 to be closely related to the *V. harveyi* strain 2–22 (99.61% similarity in the analyzed sequence of the 16S rRNA gene), the second isolate (here referred to as *Vibrio* sp. 2 to be closely related to the *Vibrio owensii* strain 2–23 (95.49%), and the third isolate (here referred to as *Vibrio* sp. 3 to be closely related to the *V. fortis strain Gumab* (96.81%). Then, 16S rRNA sequences were further analyzed against other available bacterial 16S rRNA sequences in the NCBI database to construct a phylogenetic tree (Figure 2). The analysis confirmed the results by BLAST and proposed bacterial isolate *Vibrio* sp. 1 as close to other strains of *V. harveyi* including the pathogenic *V. harveyi S0908* [40], *Vibrio* sp. 2 close to other strains of *V. owensii* including the pathogenic *V. owensii OCN002* [41], and *Vibrio* sp. 3 close to other strains of *V. fortis* including the virulent *V. fortis Gumab* [40].

### 3.2. In Vitro Antibiotic Resistance Assay

**Figure 3:**
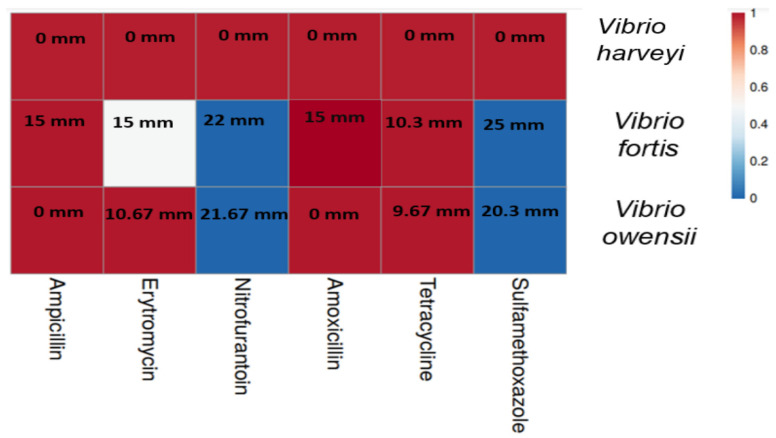
Susceptibility level of the bacterial isolates to different antibiotics. A heatmap diagram presents the susceptibility level of each of the isolated bacteria to six antibiotics common in aquaculture. Diagram colors represent the level of susceptibility as sensitive (blue), intermediate (white), or resistant (red). The value in each frame is the mean diameter (in mm) of the growth inhibition zone of the examined bacterium due to exposure to the antibiotic compound, as measured by the Kirby–Bauer disc diffusion method.

The susceptibility test confirmed the resistance of the three bacterial isolates to various antibiotics (Figure 3). Among the bacteria, *Vibrio* sp. 1. of the taxon *V. harveyi* revealed a high resistance to all six examined antibiotics. At the same time, none of these substances inhibited its growth, even slightly. All examined bacteria are defined as resistant to tetracycline, amoxicillin, and ampicillin. Nitrofurantoin and sulfamethoxazole significantly inhibited the growth of *Vibrio* sp. 2 and 3 (Figure 3). According to the measured diameter of the inhibition zone in the cultures of these two bacteria, *Vibrio* sp. 3 of the taxon *V. fortis* is considered more sensitive to the different antibiotics than *Vibrio* sp. 2 of the taxon *V. owensii*.

### 3.3. Antibiotic-Resistant Genes of Vibrio spp.

**Figure 4:**
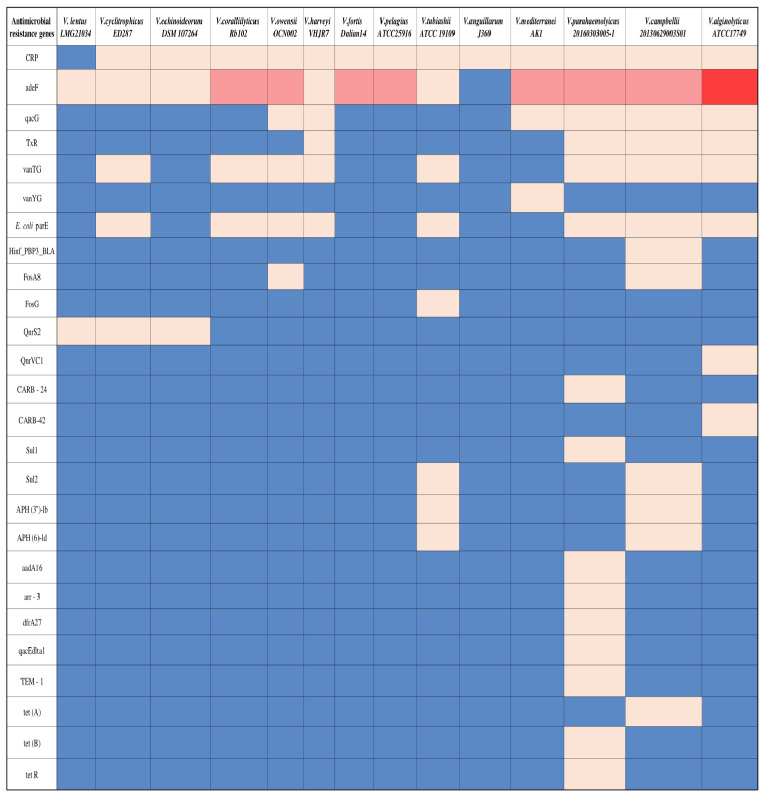
A heatmap illustrates the capacity of antibiotic resistance genes in the various examined genomes of *Vibrio* sp. Each column represents a specific examined bacterial genome. The number of copies of each of the listed ARGs in the genome is identified by color with a blue-colored square for no copies, cream for one copy, pink for two copies, or red for three copies.

The in silico analysis of 14 genomes of *Vibrio* spp. revealed a total of 26 ARGs (Table 2). The different ARGs target various drugs, including macrolides, penams, fluoroquinolones, tetracycline, glycopeptides, phosphonic acids, cephalosporin, cephamycin, sulfonamides, aminoglycosides, rifamycin, diaminopyrimidine, and cephalosporin, via various mechanisms, as described in Table 3. Among the examined bacteria, *V. parahaemolyticus 20160303005-1* is considered the richest in ARG content with 15 different ARGs, 9 of them present only in the genome of this bacterium (Figure 4). In contrast, *Vibrio anguillarum J360* contained only one copy of the CRP (C-reactive protein) gene in its genome and was considered the poorest among the examined bacteria. *V. lentus LMG21034* is also relatively poor in ARGs, having only the *adeF* and *QnrS2* genes that may provide immunity against fluoroquinolones. Among the detected genes, *CRP*, which is responsible for resistance to macrolides (e.g., erythromycin), penams (e.g., ampicillin and amoxicillin), and fluroquinolones, is the most common among the examined *Vibrio* genomes, being present in 13 of the 14 bacteria (*V. lentus LMG21034* the exception). The *adeF* gene that is responsible for resistance to fluoroquinolone and tetracycline is also shared by 13 strains, with *V. anguillarum J360* being the sole exception. Some detected antibiotic-resistant genes provide immunity against the same drug but via different mechanisms (Table 3). For example, *CRP* and *E. coli parE* genes are associated with resistance to fluoroquinolone and tetracycline, but the immune mechanism against these drugs differs. Furthermore, 50% of the examined genomes (7 out of 14 strains) included both genes in their genome. Six of the other seven genomes had only the *CRP* gene, while *V. lentus LMG21034* contained neither *CRP* nor *E. coli parE* (Figure 4).

## 4. Discussion

Aquatic antibiotic-resistant pathogens threaten the aquaculture industry by limiting the list of efficient bioactive compounds for disease control and spreading immunity against such compounds. The current research provides a first prognosis on the potential association of three isolated strains of *Vibrio* with a red spotting infection in the sea urchin *T. gratilla elatensis*. Furthermore, the in vitro susceptibility trials and in silico genomic analyses provide innovative insight into the diverse arsenal of the ARGs of these and other pathogenic *Vibrio*, emphasizing strain variability at both the genus and species level. The research underlies the complexity of controlling pathogenic *Vibrio* in mariculture facilities, including in *T. gratilla* echinocultures.

Strain variability in pathogenic *Vibrio* bacteria was evident from the in silico screening of ARGs in the bacterial genomes and the in vitro susceptibility assays. Out of 14 bacterial genomes, only 2 bacteria, *V. harveyi VHJR7* and *V. coralliilyticus RB102*, revealed a similar arsenal of ARGs, but with differences in the gene copy numbers. Consistent with the results at the genomic level, the in vitro trials also identified dissimilarity between the bacterial isolates concerning their immunity against the various antibiotics. Furthermore, we also report an inter-specific variability in antibiotic resistance. For example, the resistance to nitrofurantoin and sulfamethoxazole that was measured in the isolated *V. harveyi* was not evident in its closely related strain *V. harveyi VHJR7*, which lack genes like *oqxA* and *oqxB* for nitrofurantoin resistance or *Sul1* and *Sul2* for sulfamethoxazole resistance [62,63]. Another example is the here-isolated *V. fortis*-like bacterium, which resisted ampicillin, erythromycin, amoxicillin, and tetracycline, as compared to its close relative strain of *V. fortis dalian14* that consists of only two ARGs of the *adeF* and *CRP* that may not be sufficient for resisting aminopenicillins like ampicillin or amoxicillin. The variation in susceptibility to antibiotics at the strain level, which is reported here for *V. owensii, V. fortis*, and *V. harveyi*, has been supported by other studies that challenged different isolates with different drugs [64,65,66]. Another important aspect of studying strain variability is identifying novel resistance or susceptibility. Such can be defined when challenging a known strain with compounds that have yet to be examined or refuting a reported resistance or susceptibility. Concerning the current isolated strains, the immunity of *V. harveyi* to the different antibiotics is not novel and agrees with other studies that reported immunity against these compounds in different strains [67,68]. While results of previous studies support the here-measured resistance of the isolated *V. fortis*-like bacterium to tetracycline and ampicillin [69,70], the sensitivity to erythromycin seems to be unique to the current isolated strain as compared to other *V. fortis* bacteria that were found to be resistant to this drug [64,71]. As for the *V. owensii*-like isolate, the here-measured immunity against tetracycline, erythromycin, amoxicillin, and ampicillin has been reported in other strains of this taxon [72,73]. Still, the identified sensitivity to sulfamethoxazole seems unique to the here-isolated strain since other studies reported resistance to this drug by various strains of *V. owensii* [69,73,74]. Moreover, susceptibility to sulfamethoxazole, which was found here in both the *V. fortis* and *V. owensii*-like isolates, can be considered rare in the *Vibrio* taxon, as demonstrated by the collection of at least 315 strains of *Vibrio* from various wastewater plants in South Africa and Nigeria that were found to be resistant to this drug [75,76].

Even though we have evident strain variability, our in vitro and in silico data also propose a core resistance to tetracyclines and penams like amoxicillin and ampicillin. The resistance to these compounds is widespread in *Vibrio* bacteria [26,77] and may be associated with the presence of the *adeF* and *CRP* genes in the genome [73]. The presence of *adeF* and *CRP* as core genes can induce resistance to other bioactive compounds of the fluoroquinolones, such as ciprofloxacin and gemifloxacin, but such bioactive chemicals are not as common as tetracyclines for disease-control in aquaculture [73]. While the three bacterial isolates share resistance to other antibiotics like ampicillin, erythromycin, and amoxicillin, their close relatives may only share resistance to erythromycin derived by the presence of the *CRP* gene, which induces immunity to macrolides [78]. Interestingly, the current research highlights a more remarkable similarity in antibiotic resistance between the different isolated strains of *Vibrio* than between the different species for which genomes were analyzed. We assume that the shared immunity against multiple drugs by the isolated strains is due to their shared origin of the red-spotting-infected tissue of sea urchins in the echinoculture. Notably, various studies confirmed that aquaculture systems are a great source of bacteria with a shared resistance to multi-drugs. One example is a shared resistance to ampicillin, erythromycin, sulfamethoxazole, tetracyclines, oxytetracycline, and chloramphenicol that was identified in isolates of *Vibrio fluvialis, V. parahaemolyticus*, and *V. vulnificus* during four independent samplings in four fishpond effluents in Benin City, Nigeria [79]. A similar phenomenon has been evidenced recently in a seafood market in Italy where Vibrio isolates from purchasable mussels shared immunity against various drugs, although their host mussels reached the market from different farms in the area [80]. These phenomena highlight the role of HGT in the aquaculture environment in providing immunity against various drugs to the resident *Vibrio* bacteria, including the aquaculture pathogens within this taxon. Although bacterial genomes are preserved over a short scale of time, i.e., from one generation to the following [81], plasticity, such as the acquired immunity against drugs, can be achieved over the longer run, i.e., throughout several generations, through horizontal gene transfer, mutations, and the transfer of mobile genetic elements [82,83]. One reason for this is the fact that the set conditions in the aquaculture facility, like the temperature, pH, and aerobic environment, favor not only the cultured species, but also support the succession of pathogenic and non-pathogenic *Vibrio* [84], which can further distribute antibiotic resistance in this environment through HGT. Diseases and the administration of antibiotics stimulate pathogen succession, expanding antibiotic resistance within the local microbial community, including between pathogens in the community [85,86]. That said, our study supports the claim that the aquaculture environment accelerates genomic modifications toward immunity against multiple drugs shared by various local bacteria. We assume that the development of immunity against any particular antibiotic is associated with the exposure of the bacterium to this drug or a bacterium that carries and spreads the required genetic elements for resistance. This can also explain the core- and strain-unique resistance, or susceptibility, within members of the *Vibrio* taxon.

The current study seems to be the first to report red spotting in *T. gratilla* sea urchins, the potential causative agents, and their immunity against various antibiotics. This knowledge is essential for decision making concerning the efficient treatment for eliminating such bacteria in echinocultures if needed. *T. gratilla* possesses a high commercial value due to its rapid growth and high nutritional value [87]. Despite its importance in seafood markets, reports on diseases that harm this species are relatively rare and seem to include only the lesions syndrome [51,88] and the bald sea urchin disease [89]. While not documented previously in *T. gratilla*, red spotting disease has been reported in another valuable sea urchin, *S. intermedius*, in nature and echinoculture [1,2], causing severe damage with a mass mortality of 90% of the culture [2].

*Vibrio* bacteria’s reputation for causing disease encompasses various invertebrates and vertebrates, including sea urchins. Among the diseases associated with the taxon in sea urchins are vibriosis, black peristomial, bald sea urchins, lesion syndrome, spotting, and red spotting. *V. splendidus* is probably the most infamous taxon, associated with five diseases, followed by *V. harveyi* and *V. fortis*, which were associated with both the lesion syndrome and spotting disease [35,51] in *S. intermedius* and now in red spotting in *T. gratilla* in the current study. An interesting phenomenon is the co-occurrence of these two bacteria in samples from the infected tissue of the sea urchins that were examined in these four independent studies, while none of the sea urchin diseases surveyed here reported on the isolation of only one of those two taxa and not the other. When examined for virulence, *V. fortis* or *V. harveyi* negatively affected naïve animals [35,40]. We assume that the co-occurrence of *V. fortis* and *V. harveyi* is not by chance and may hint that both harm the sea urchin while moribund, including in the red spotting of *T. gratilla* in the current research. Identifying these two bacteria, as well as *V. owensii*, as potential pathogens of the red spotting in *T. gratilla* is novel, as this disease has received less attention in research on sea urchins in general and on *T. gratilla* specifically. The fact that none of the three isolates were detected in non-infected individuals or in healthy tissues strengthens our assumption regarding their association with this disease in the echinoculture. The here-reported data on the resistance to multiple drugs by these isolates are essential for developing protocols for their elimination from red-spotting-infected echinocultures and their prevention via prophylactic treatments. Concerning the in vitro and genomics analyses, we propose that only nitrofurantoin and sulfamethoxazole may be helpful against *V. fortis* and *V. owensii*-like pathogens, but not against the *V. harveyi*-like one. The immunity of the isolated *V. harveyi* to sulfamethoxazole should be taken seriously into consideration since inheriting resistance to this antibiotic is likely through the horizontal transfer of integrating conjugative elements such as SXT, which carries multiple resistance genes against sulfamethoxazole, streptomycin, and trimethoprim [90]. On the other hand, the development and conferring of resistance to nitrofurantoin has been proposed to be relatively slow through mutations in genes like nfsA, nfsB, and ribE [91]. Therefore, using nitrofurantoin in echinocultures with red spotting outbreaks seems reasonable in parallel to studies of other drugs against these potential pathogens.

## 5. Conclusions

To conclude, the current research proposes three strains of *Vibrio* as associated with the red spotting infection in the sea urchin *T. gratilla elatensis*. Unfortunately, yet not surprising, all three strains exhibit multi-drug resistance which may enable them to withstand the common treatments with antibiotics in echinocultures. In agreement with the knowledge that was gained here from the genomic survey of harmful pathogens of the *Vibrio*, the here-reported strains also show a different profile of antibiotic resistance for each. Thus emphasizing the variability at both the genus and species level and the difficulty in disease controlling when caused by *Vibrio*.

## Figures and Tables

**Table 1:** *Vibrio* bacteria associated with sea urchin diseases.

Disease Name	Infected Sea Urchin	Proposed Pathogen	References
Black peristomial disease	*Strongylocentrotus intermedius*	*Vibrio splendidus* *Vibrio lentus* *Vibrio atlanticus* *Vibrio echinoideorum*	[33]
Lesion syndrome/Tissue necrosis	*Strongylocentrotus droebachiensis*	*Vibrio echinoideorum*	[34]
	*Strongylocentrotus intermedius*	*Vibrio splendidus* *Vibrio fortis* *Vibrio shilonii* *Vibrio harveyi*	[35]
Bald sea urchin disease	*Strongylocentrotus purpuratus*	*Vibrio anguillarum*	[36]
	*Paracentrotus lividus*	*Vibrio splendidus*	[37]
Vibriosis	*Holopneustes purpurascens*	*Vibrio anguillarum*	[38]
	*Heliocidaris erythrogramma*		
	*Archaeopneustes hystrix*	*Vibrio alginolyticus*	[39]
	*Paleopneustes cristatus*		
Spotting disease	*Strongylocentrotus intermedius*	*Vibrio shilonii* *Vibrio splendidus* *Vibrio harveyi* *Vibrio fortis*	[40]
		*Vibrio owensii* *Vibrio aquaticus*	[41]
Red spotting disease	*Strongylocentrotus intermedius*	*Vibrio coralliilyticus*	[1]
	*Tripneustes gratilla*	*Vibrio harveyi* *Vibrio fortis* *Vibrio owensii*	Current study

**Table 2:** *Vibrio* spp. examined in the in silico survey. The table provides data on the examined strains’ identity, origin, and associated diseases.

Bacterial Strain and Sequence Accession Number in the Genbank Database	Infected Animal	Associated Disease	References
*Vibrio fortis Dalian 14* *Accession no. GCF_000695685.1*	Sea urchin *Strongylocentrotus intermedius*	Lesion syndrome	[41,49]
*Vibrio coralliilyticus Rb102* *Accession no. GCF_029541605.1*	Sea urchin *Strongylocentrotus intermedius*	Red spotting disease	[1]
*Vibrio echinoideorum DSM 107264* *Accession no. GCF_024347455.1*	Sea urchin *Strongylocentrotus droebachiensis*	Lesion infection	[50]
*Vibrio cyclitrophicus ED287* *Accession no. GCF_023206055.1*	Sea urchin *Strongylocentrotus intermedius*	Red spotting disease	[51]
*Vibrio lentus LMG21034* *Accession no. GCF_024347555.1*	Sea urchin *Strongylocentrotus purpuraus*	Vibriosis	[52]
*Vibrio harveyi VHJR7* *Accession no. GCF_000442925.1*	Fishes Seabass *Lates calcarifer* Humpback grouper *Cromileptis altivelis* Black tiger shrimp *Penaeus monodon*	Vibriosis	[53]
*Vibrio owensii OCN002* *Accession no. GCF_000818275.1*	Coral *Montipora capitat*	White Syndrome	[54]
*Vibrio alginolyticus ATCC17749* *Accession no. GCF_000354175.2*	Sea cucumber *Holothuria atra*	Skin ulceration disease	[55]
*Vibrio mediterranei AK1* *Accession no. GCF_000181535.1*	Coral *Oculina patagonica*	Bleaching	[56]
*Vibrio parahaemolyticus 20160303005-1* *Accession no. GCF_009883875.1*	Shrimp (larvae) *Penaeus vannamei*	Glass post-larval disease	[57]
*Vibrio campbellii 20130629003S01* *Accession no. GCF_002140055.1*	Shrimp *Litopenaeus vannamei*	Acute hepatopancreatic necrosis disease (AHPND)	[58]
*Vibrio anguillarum strain J360* *Accession no. GCF_003399575.2*	Lumpfish *Cyclopterus lumpus*	Vibriosis	[59]
*Vibrio tubiashii ATCC 19109* *Accession no. GCF_000772105.1*	Larval shellfish *Crassostrea virginica* and *Crassostrea gigas*	Bacillary necrosis	[60]
*Vibrio pelagius ATCC25916* *Accession no. GCF_024347575.1*	Turbot fish (larvae) *Scophthalmus maximus*	Swelling and necrosis of gill secondary lamellae, intestinal mucosa, and tissue necrosis	[61]

**Table 3:** Antimicrobial resistance genes, targeted drugs, and resistance mechanisms. The list consists of genes identified in the *Vibrio* pathogens’ examined genomes.

Antimicrobial Resistance Genes	Drug Class	Resistance Mechanism
*CRP*	Macrolides, fluoroquinolones, penams	Antibiotic efflux-Intracellular pump expels antibiotics.
*adeF*	Fluoroquinolones, tetracyclines	
*qacG*	Disinfecting agents and antiseptics	
*TxR*	Tetracyclines	
*FosA8*	Phosphonic acids	
*qacEdlta1*	Disinfecting agents and antiseptics	
*tet (A)*	Tetracyclines	
*tet (B)*	Tetracyclines	
*tet R*	Tetracyclines	
*tet R*	Tetracyclines	Antibiotic target alteration-Genetic modification disrupts antibiotic target sites.
*vanTG*	Glycopeptides	
*vanYG*	Glycopeptides	
*E. coli parE*	Fluoroquinolones	
*Hinf_PBP3_BLA*	Cephalosporin, cephamycin, penams	
*dfrA27*	Diaminopyrimidines	Antibiotic target replacement-Substitution of antibiotic targets occurs.
*Sul1*	Sulfonamides	
*Sul2*	Sulfonamides	Antibiotic target protection-Molecular shielding safeguards antibiotic target sites.
*QnrVC1*	Fluoroquinolones	
*CARB-24*	Penams	Antibiotic inactivation-Chemical alteration disabling antibiotics’ effectiveness.
*CARB-42*	Penams	
*APH (6)-ld*	Aminoglycosides	
*aadA16*	Aminoglycosides	
*arr-3*	Rifamycins	
*TEM-1*	Monobactams, cephalosporins, penams	
*FosG*	Phosphonic acids	

## Data Availability

The authors declare that all of the data that were used in this research are available and will be provided upon request due to privacy or ethical restrictions.
